# Printed dose-recording tag based on organic complementary circuits and ferroelectric nonvolatile memories

**DOI:** 10.1038/srep13457

**Published:** 2015-08-26

**Authors:** Tse Nga Ng, David E. Schwartz, Ping Mei, Brent Krusor, Sivkheng Kor, Janos Veres, Per Bröms, Torbjörn Eriksson, Yong Wang, Olle Hagel, Christer Karlsson

**Affiliations:** 1 Palo Alto Research Center, Palo Alto, CA 94304, USA; 2 Thin Film Electronics AB, Linköping, Sweden

## Abstract

We have demonstrated a printed electronic tag that monitors time-integrated sensor signals and writes to nonvolatile memories for later readout. The tag is additively fabricated on flexible plastic foil and comprises a thermistor divider, complementary organic circuits, and two nonvolatile memory cells. With a supply voltage below 30 V, the threshold temperatures can be tuned between 0 °C and 80 °C. The time-temperature dose measurement is calibrated for minute-scale integration. The two memory bits are sequentially written in a thermometer code to provide an accumulated dose record.

Solution printing processes are well-suited for high-throughput, low-cost fabrication on flexible substrates. While tiny discrete CMOS chips have been incorporated into electronics systems on flexible substrates, the conventional solder-reflow bonding process for discrete chips typically requires high temperatures incompatible with polyethylene-based substrates. Print fabrication can be performed at lower temperatures, enabling the use of these lower-cost materials. Printing is also capable of covering large areas economically. Thus printed electronics are compelling for internet-of-things applications that require a large volume of devices, such as sensor networks and smart packaging. Recent advances in organic electronic circuits have demonstrated that sensing^1^,^2^,^3^,^4^, signal processing and conditioning^5^,^6^,^7^,^8^, and communications^9^,^10^ functionalities can be realized with solution-processed materials. Here we report on the development of an additively-printed organic circuit platform capable of processing sensor data and recording dose measurements to nonvolatile memories^11^,^12^,^13^ in an integrated tag. The tag demonstrates a system application for the case of time-temperature dose measurement, which can be used to track hazardous conditions or quantify spoilage in perishables. While previous examples of organic temperature sensing systems^14^,^15^ have focused on detecting a critical temperature threshold, there is a need to sense time-temperature dose (TTD) values to account for both critical temperature and exposure time limits. A TTD is a temperature-dependent time interval and can be measured, for example, to monitor spoilage affected by incubation temperature *and* duration. Moreover, the circuit platform can be generalized to measure other time-integrated sensor signal “doses”, for example, by replacing the thermistor with other resistive sensors such as strain gauges or chemical sensors, the printed circuits are potentially applicable to a variety of sensing applications.

One of the key features of this demonstration is the integration of multiple printing technologies and device types, including circuitry designed according to the constraints of an all-additive inkjet organic thin-film-transistor (OTFT) process, including the use of as few OTFTs as possible to maximize yield. Moreover, the limitations of organic circuits call for a design approach that focuses on utilizing the simplest circuit possible for a given application. For example, in this design, the tight matching tolerance required by a differential comparator would make it difficult to implement. In contrast the chosen inverter-based threshold circuit allows larger tolerance and is more suitable for this system. A significant challenge in the design is to achieve minute-scale integration times. Approaches such as multiplying integration time with a digital counter are not desirable because they require a substantial increase in the number of OTFTs. It is preferable to directly integrate charge for the full target interval, as is done here. A current signal generated by the sensor is integrated on a capacitor. When the integrated charge reaches a threshold, the circuit writes to a memory cell. The memory bits are sequentially written to form a thermometer-coded output. Each bit in the memory array represents a certain amount of charge, *Q*, and the total number of bits written, *m,* indicates the total amount m*Q of integrated charge and the corresponding dose.

## Methods

In comparison to conventional circuits, the variance and bias-stress stability of OTFT circuits are major concerns. Device-to-device variability is incorporated into SPICE simulation models using process corners^16^, to model the typical, minimum, and maximum values of device current, which range from 0.33 to 2.0 times the typical value. Simulations are performed under each process corner to optimize sizing in OTFTs, resulting in the choices shown in the circuit schematic in Supplemental Figure S1. The changes in current and threshold voltage due to bias stress in operation are smaller than those associated with device-to-device variation in the existing OTFT process (Supplemental Figure S3). Hence the process corners are sufficient to account for the temporal variation due to bias stress.

In this prototype the components are fabricated on discrete plastic polyethylene naphthalate foils, which are bonded to a screen-printed silver interconnect substrate and connected by conductive silver ink. The transistor circuits are printed in sections to facilitate iteration, testing, and integration as shown by the dashes in the circuit schematic of Supplemental Figure S1. In production, these circuit blocks could be combined, for example, in a roll-to-roll process. The screen-printed thermistor divider, provided by PST Sensors, comprises a silicon-nanoparticle thermistor and carbon resistor, both with nominal resistances of 50 MΩ at 25 °C. The nonvolatile memories are 80 μm × 80 μm ferroelectric capacitors patterned by gravure printing on polyethylene terephthalate foils. The memory dielectric is a PVDF copolymer thin film. For the Ta_2_O_5_ capacitors, the dielectric film is processed in ambient conditions by anodization of Ta gate metal at 75 V in a boric acid solution (0.4% by weight, adjusted to *p*H = 7 with NaOH) resulting in 400 nm Ta_2_O_5_ film. The complementary OTFT process is chosen from among available design styles, because complementary circuits^17^,^18^,^19^ consume less power than unipolar circuits, use fewer OTFTs than pseudo-CMOS circuits^20^, and require fewer process steps than dual-gate circuits^21^. Both p-channel^22^,^23^ and n-channel^24^,^25^,^26^ OTFTs are top-gate devices fabricated by inkjet printing, with average mobility of 0.1 cm^2^ V^−1^ s^−1^. The circuit design is based on a transistor channel length of 35 ± 5 μm. The materials, structure, typical current-voltage characteristics of the printed OTFTs are shown in supplemental Figures S2 and S3.

## Results

**Figure 1:**
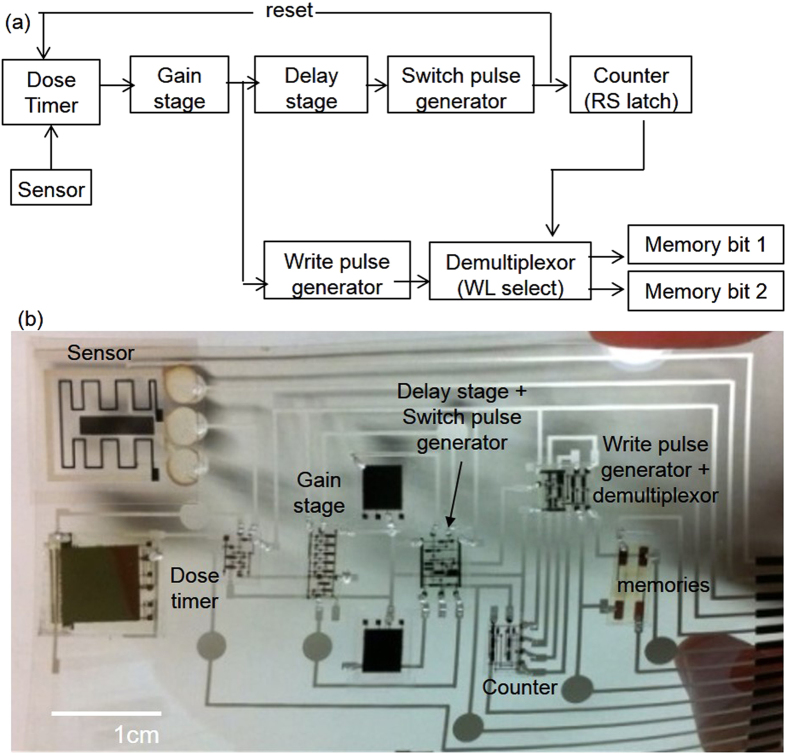
(**a**) Block diagram of the dose-recording circuit, and (**b**) photograph of the integrated tag.

The printed TTD measurement tag comprises a thermistor divider, complementary circuits (44 OTFTs in total), and two nonvolatile memory cells. The integrated sensor current triggers the sequential writing of the memory cells, which store a thermometer-coded record of zero, one, or two TTDs. The integrator circuit is reset between measurements. The block diagram and the photograph of the integrated tag are shown in Fig. 1.

**Figure 2:**
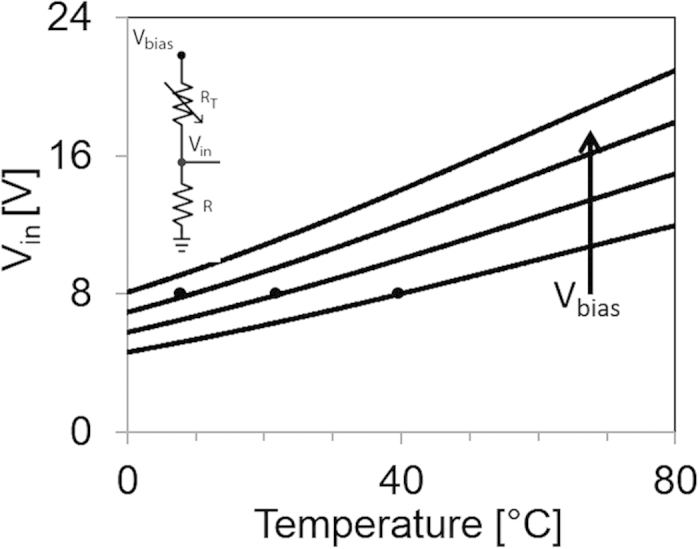
Thermistor divider with V_bias_ ranging from 20 V to 35 V, in increments of 5 V. The trigger threshold of the following stage is set at 8 V as indicated by the black dots. The corresponding threshold temperature is dependent on V_bias_. Applying higher V_bias_ leads to lower threshold temperature.

The thermistor divider provided by PST Sensors consists of a silicon-nanoparticle thermistor and a carbon resistor. The thermistor shows a temperature coefficient of −1 MΩ/°C. As the thermistor resistance falls with increasing temperature, the divider output voltage *V*_*in*_ rises as is shown in Fig. 2. Due to the large variability in devices fabricated with printing processes, the sensor and OTFT circuits require calibration to adjust the bias voltage *V*_*bias*_ on the sensor to match the trigger threshold in the signal processing circuit. The temperature threshold of the tag can range from 0 °C to 80 °C and is calibrated by changing *V*_*bias*_ such that *V*_*in*_ matches the trip voltage of the gain stage at the desired minimum trigger temperature. For example, a bias of 20 V corresponds to a 40 °C threshold. With a 30 V bias, the thermistor divider output changes by approximately 0.1 V/°C and is linear near room temperature. The printed trigger circuits are stable to within 0.1 V during 10 minutes of continuous operation in laboratory conditions. Thus the tag has the potential to achieve ±1 °C accuracy after calibration and with intermittent recovery periods to reduce bias drift. This calibration accounts for the change in transistor characteristics with temperature during the measurement. However, additional studies are needed to determine the circuit’s long-term temperature stability.

For TTD measurements, the thermistor divider is combined with the integrating RC stage. To first order, the integration time is determined by the capacitor *C*_*Timer*_ and the off-resistance *R*_*off*_ of OTFT *M*_*R*_. With a typical *R*_*off*_ value of 2 GΩ, the capacitor must have high capacitance value (>30 nF) and low leakage (<10 nA) to realize an RC delay of one minute. To meet these requirements, a 400-nm anodized Ta_2_O_5_ high-k dielectric film with a measured capacitance of 50 nF cm^−1^ is used for the capacitors. The tag has been operated with integration times ranging from 10 s (with *R*_*off *_= 0.25 GΩ) to 400 s (*R*_*off *_= 10 GΩ) by varying the gate bias on *M*_*R*_, with *C*_*Timer*_ = 40 nF. For an alternative TTD circuit, the thermistor *R*_*T*_ can be directly connected to *C*_*Timer*_ for the integrating stage. However, this option is not implemented for minute-scale integration, because such a solution would require extremely high thermistor resistance values on the order of 1 GΩ, and would be more sensitive to their accuracy.

**Figure 3:**
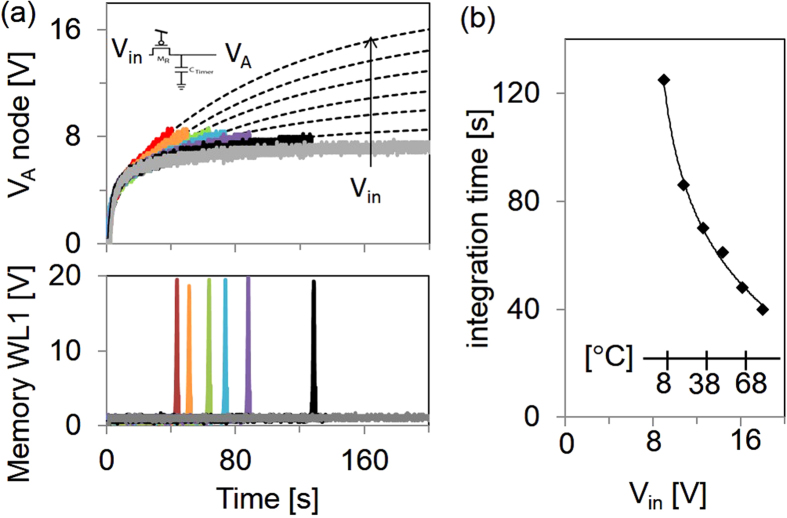
(**a**) Signals at node V_A_ and WL1 as a function of time and V_in_, where V_in_ varies from 7.2 V to 18.0 V in increments of 1.8 V. The dotted lines are based on double exponential fits. (**b**) The circuit integration time as a function of V_in_. The V_in_ axis is converted to a temperature scale (inset), for an instance with thermistor divider calibrated to V_bias _= 30 V.

As the integration capacitor is charged (Fig. 3), the voltage at node *V*_*A*_ rises faster with higher

*V*_*in *_= *V*_bias_*R/(*R*_*T *_+ *R*). As *V*_*in*_ is increased, it takes less time to reach the trip voltage of the circuit set at 8 V, as indicated by the timing of the output pulses on memory wordline *WL*. With an input below the set point, for example *V*_*in *_= 7.2 V, the output pulse will not be triggered. Above the set point, the trigger time is a function of *V*_*in*_. With *V*_*in *_= 18 V, a pulse is generated at time *t *= 40, whereas with *V*_*in *_= 9 V, the pulse is at *t *= 130 s. This characteristic allows setting a dose threshold that is dependent on both the duration and the magnitude of an input signal. The *V*_*A*_ voltage does not have a pure RC exponential characteristic, but can be fit with a double-exponential time dependence, according to 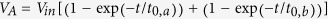, with *t*_*0,a *_= 10 s and *t*_*0,b *_= 150 s. This is the result of non-linear channel resistances of M_Reset_ and M_R_, as well as electrical bias stress. The integration time *t*_*integration*_ with respect to *V*_*in*_ is shown in Fig. 3(b), and this data fits the natural logarithmic equation *t*_*integration *_= 36.2 ln (*V*_*in*_−8) +124.8. The inset axis of Fig. 3(b) shows the temperature correspondence of *V*_*in*_ with the thermistor divider at V_bias _= 30 V. From this calibration, one can infer that, for example, if the sample is held at 37 °C for longer than 70 s, or at 66 °C for longer than 50 s, the circuit will trigger and record the TTD into a memory cell.

**Figure 4:**
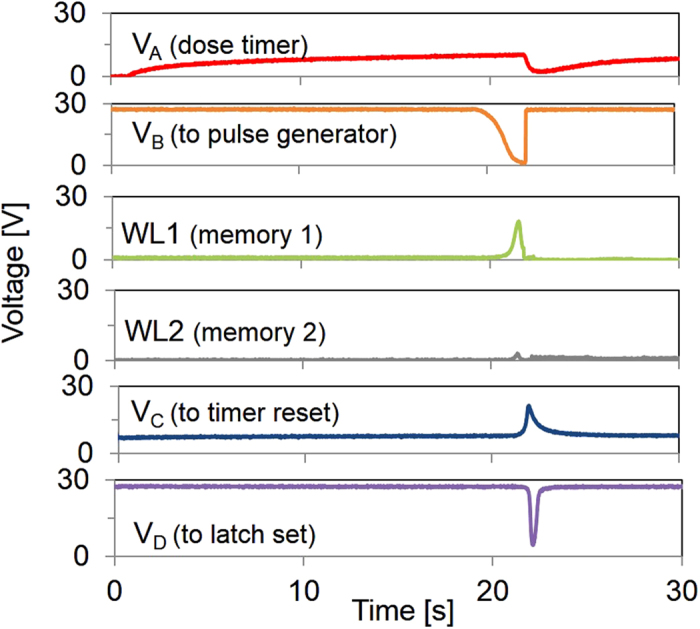
Measured signals at the circuit nodes indicated in Supplemental Figure S1 with input voltage Vin = 20 V and supply voltage VDD = 28 V.

Figure 4 shows an overview of the tag signals during operation with a supply voltage of *V*_*dd *_= 28 V, and the circuit nodes as labeled in Supplemental Figure S1. When *V*_*A*_ reaches the trip point of the gain stage at t = 20 s, the gain stage sends a falling edge to node *V*_*B*_. This node is connected to two pulse-generator circuits, one for writing to the memory word lines (*WL1, WL2*), and the other for generating pulses at *V*_*C*_ to reset the integrator and at *V*_*D*_ to set the RS latch, which addresses the memory and is reset at start-up. With a larger memory array, a shift register and a demultiplexor could be used instead of the RS latch. The pulse generators are NOR-based monostable multivibrators. Two pulse generators with a delay between the respective signals are used to accommodate the required delay of the control signals, ensuring that the first memory cell is fully written before the RS latch is set, as well as to satisfy the different requirements of the reset and memory-write pulses. Even with the augmented on/off ratio provided by the inverter in the reset circuit, a wide pulse (realized by the addition of *C*_*Slow *_= 6 nF) is needed to ensure discharge of the *C*_*Timer*_ capacitor to ground and reset the integrator. The pulse widths are also dependent on the slope of the input edge, which is shallow enough that the average pulse width is on the order of 1 s. The pulse waveform generated by the write-pulse generator is used to write memory cells; for data retrieval, the nonvolatile memory cells are read with an external charge amplifier reader unit, following the procedure in Ref. 16. If a pulse with voltage magnitude above 17 V is applied to a memory cell, it is written to state “0”, which appears at the reader output as the large amplitude waveform in supplemental Figure S4(c). However, if there is no pulse or if the pulse amplitude is below 5 V as in the case of a disabled word line (e.g., *WL2* in Fig. 4), the memory bit remains in its default state “1”, corresponding to the relatively lower amplitude waveform in supplemental Figure S4(d).

**Figure 5:**
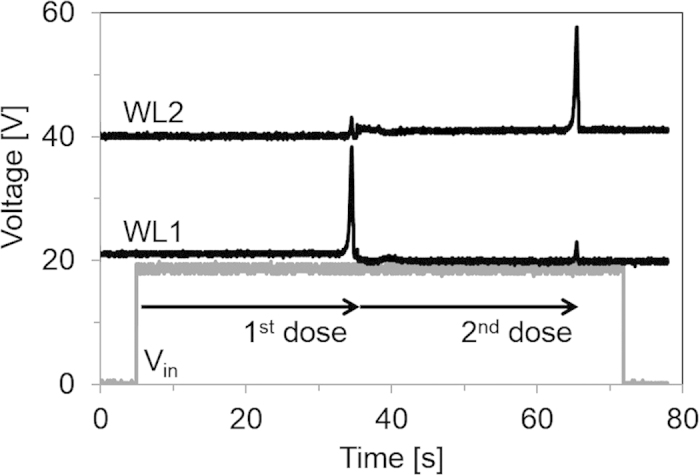
Sequential writing of two memory bits, with supply voltage V_DD _= 28 V. WL1 and WL2 are offset by 20 V and 40 V, respectively.

In Fig. 4 while a pulse is passed to *WL1*, *WL2* is maintained below 3 V, and the second memory cell is not written. When the RS latch is set after the first memory bit has been written, *WL1* is disabled, and *WL2* is enabled to address the second memory cell. Figure 5 shows the *WL1* and *WL2* waveforms during a long exposure event equivalent to two doses. The sequential writing of memory cells demonstrates that the thermometer-code recording method is successfully implemented. During initialization, the memory cells are set to state “1”. In the inverted thermometer encoding used, when there is no threshold event, the two memory cells remain in the default state and the readout result is “11”. Upon the first dose event, the first cell switches state and the 2-bit readout will be “01”. After the second dose, the readout will be “00”. Thus, three combinations of the two-bit memory are possible. The combination “10” is not valid as the second cell is never written without the first cell having been previously written. The two written memory cells provide an accumulated record of events of exposure of the sample to conditions above the dose limit. Moreover, if *V*_*in*_ decreases during the course of a measurement, as by a reduction in temperature, the duration of this disruption is accounted for in the total integration time (supplemental Figure S5). High supply voltage was used in this demonstration, but recent results in using high-k dielectrics^19^ for printed OTFTs will enable the dose tag to operate at a decreased supply voltage in the future.

## Conclusions

We have demonstrated a printed sensor platform that monitors minute-scale-duration temperature doses and records the dose events in two thermometer-coded nonvolatile memory cells. This integrated organic electronic system uses a low-transistor-count design to boost yield for practical print fabrication on flexible plastic foils. If integrated with an appropriate power supply, the tag could operate as a standalone system, with nonvolatile memory for later readout. Threshold temperatures can be calibrated between 0 °C and 80 °C, covering a wide range of applications in food and medicine spoilage. In addition to temperature sensing, the system is compatible with other current-based sensors, and, by scaling up the memory addressing circuit, it can be readily extended larger memory arrays to store more data. The development of this tag is a promising step towards economical printed sensor systems.

## Additional Information

**How to cite this article**: Ng, T. N. *et al.* Printed dose-recording tag based on organic complementary circuits and ferroelectric nonvolatile memories. *Sci. Rep.* **5**, 13457; doi: 10.1038/srep13457 (2015).

## Supplementary Material

Supplementary Information
