# Supplementary material for: Printed dose-recording tag based on organic complementary circuits and ferroelectric nonvolatile memories
Source: Sci Rep. 2015 Aug 26;5:13457. doi: 10.1038/srep13457 (PMC4549707; doi:10.1038/srep13457)
Supplement: Supplementary Information [file srep13457-s1.pdf]

Supplement to printed dose-recording tag based on organic complementary circuits and ferroelectric nonvolatile memories

Tse Nga Ng*, David E. Schwartz*, Ping Mei, Brent Krusor, Sivkheng Kor, Janos Veres

Palo Alto Research Center, Palo Alto, CA 94304, USA

Per Bröms, Torbjörn Eriksson, Yong Wang, Olle Hagel, Christer Karlsson

Thin Film Electronics AB, Linköping, Sweden

*Corresponding authors: tnng@parc.com; David.Schwartz@parc.com

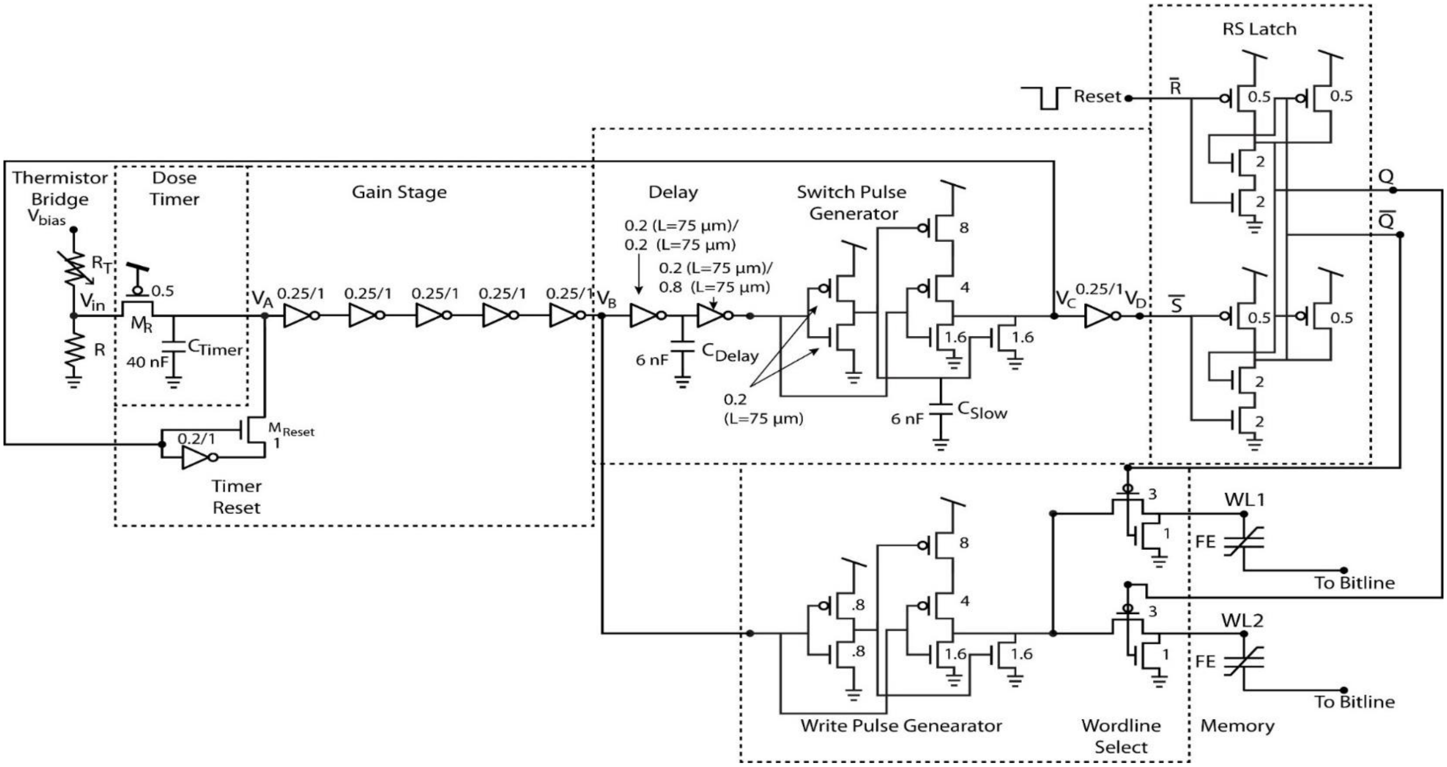

Figure S1. Schematic of the dose-recording circuit. The values next to the components indicate transistor channel widths in mm, while the transistor channel length is 35± 5 μm.

The organic thin film transistors (OTFTs) are fabricated on plastic polyethylene naphthalate foils. The electrodes of the OTFTs are inkjet printed using Ag nanoparticle dispersion. Organic semiconductors were inkjet printed to form p-channel[1] and n-channel[2] transistors. The gate dielectric is a spin-coated bilayer[3] with thickness of ~900 nm, comprised of a Teflon layer next to the semiconducting layer and a high-k PVDF-TrFE-

CTFE relaxor polymer. The vias were drilled by laser ablation.

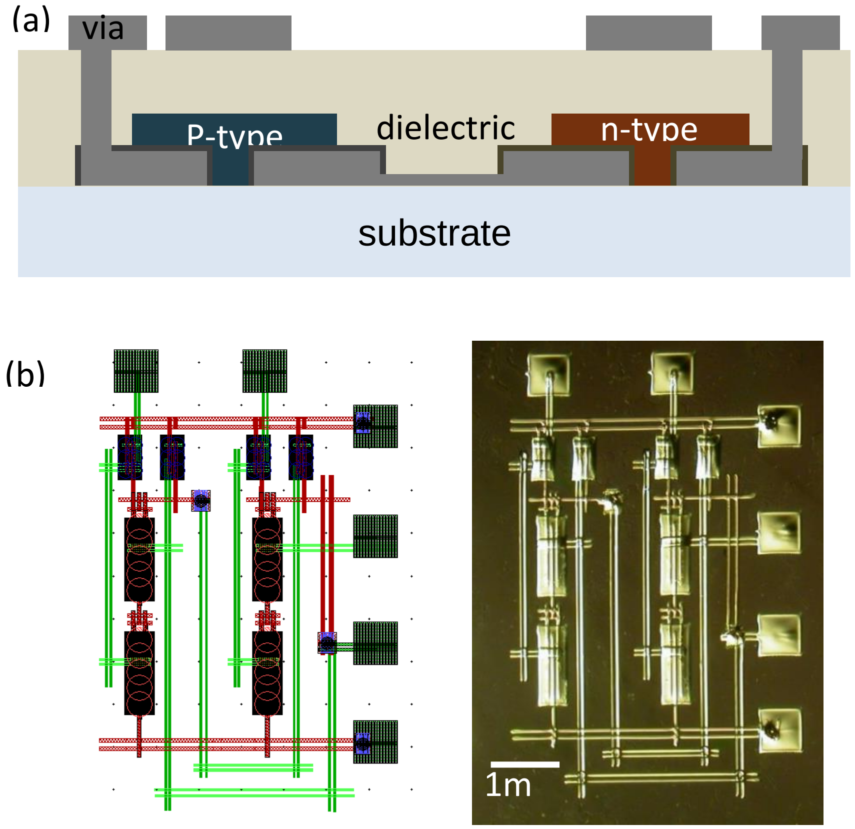

Figure S2. (a) Cross-section view of the printed transistor structures. (b) Example of a digital layout (left) and the corresponding physical circuit (right).

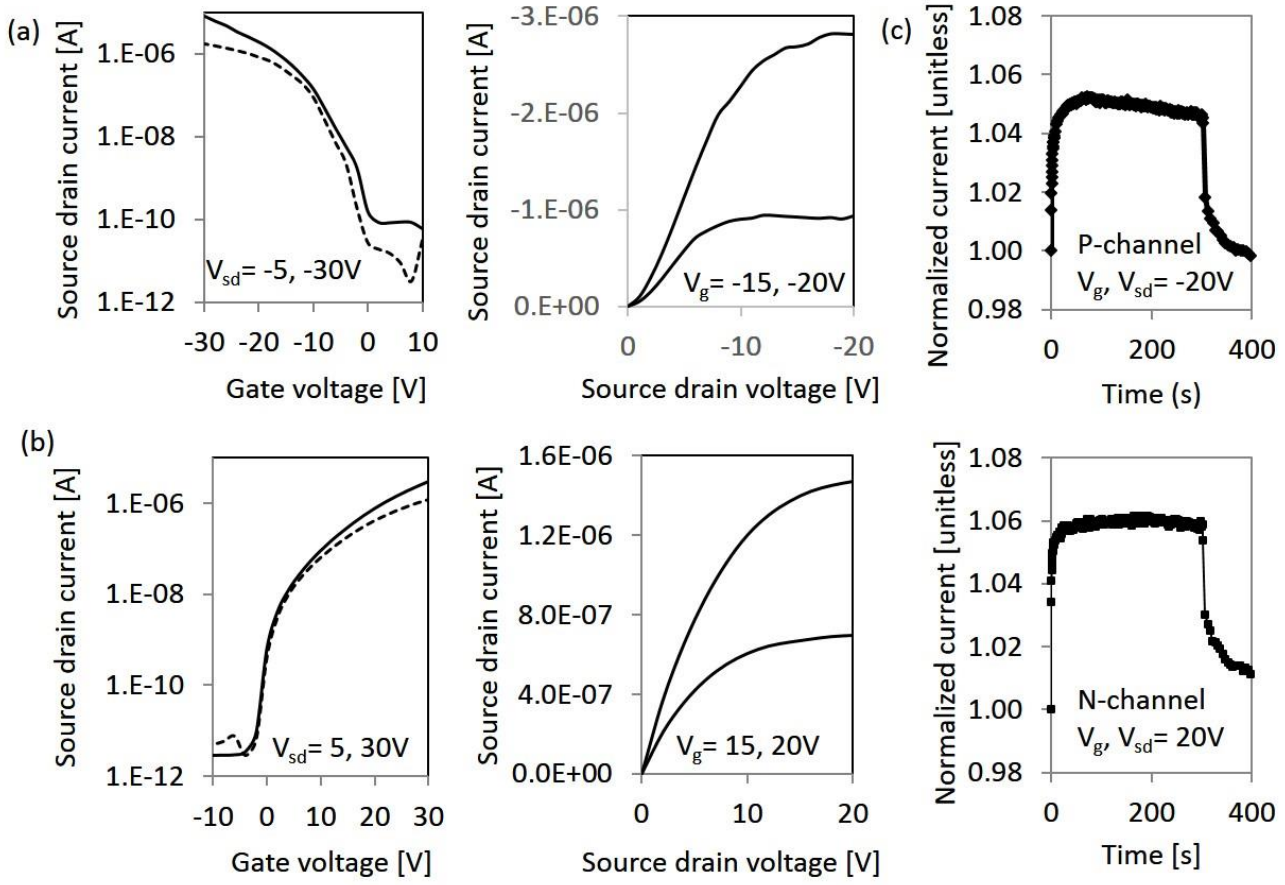

	P-OTFT mobility [cm^2/(Vs)]	P-OTFT threshold voltage [V]	N-OTFT mobility [cm^2/(Vs)]	N-OTFT threshold voltage [V]
Mean	0.12	-1.43	0.13	3.93
Max	0.21	-0.51	0.27	5.18
Min	0.06	-2.87	0.04	2.40
Std dev	0.05	0.58	0.06	0.70

Figure S3. Typical I-V characteristics of the printed transistors. Here the channel width is 1.1 mm, channel length is 35 µm, and gate capacitance is 5 nF/cm^2. (a) P-channel. (b) N-channel. The table shows the mobility and threshold voltage of 25 OTFTs for each type of semiconductor. (c) Bias stress during continuous operation (0 s to 300 s) and during pulsed gate bias at 1Hz with 5% duty cycle (300 s to 400 s). The rise in current during the first 30 s of operation is caused by slow polarization of the PVDF terpolymer high-k relaxor dielectric; thereafter the current decreases gradually.

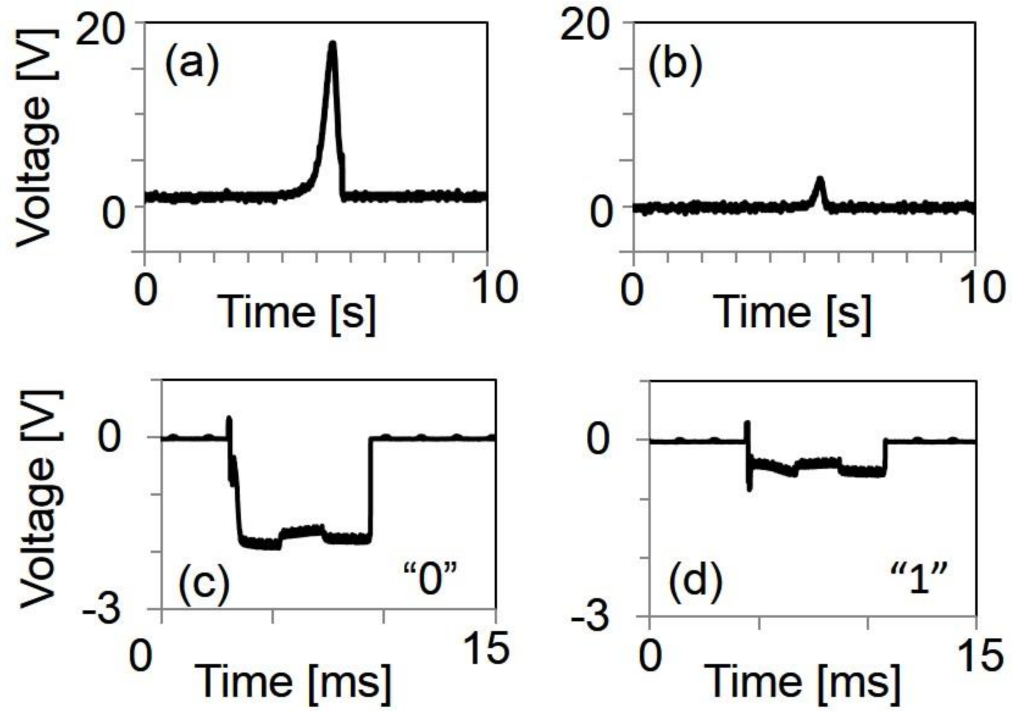

Figure S4. Waveforms of writing and reading memory. Active word line pulse (a) writes a "0", and then the external reader obtains an output "0" as shown in (c). Inactive word line waveform (b) leaves an undisturbed preset "1", as indicated by the reader output in (d).

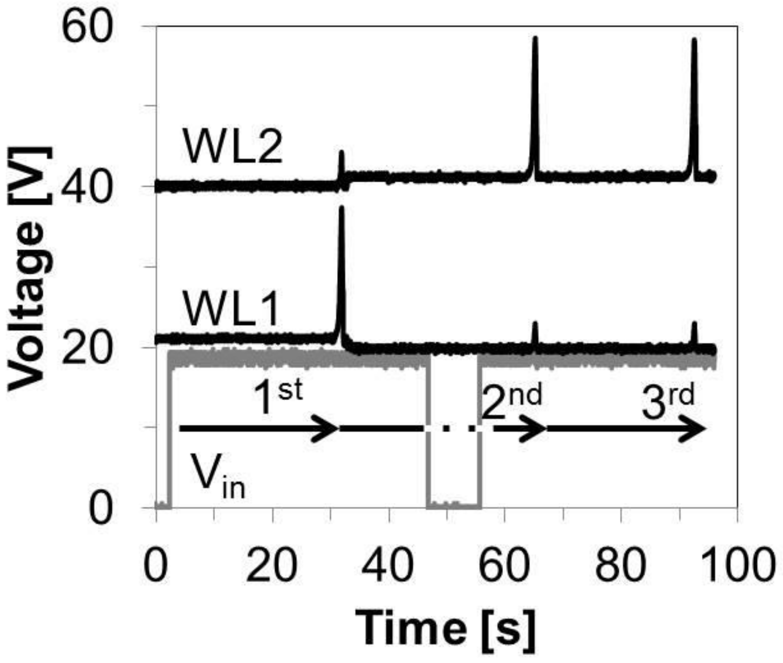

Figure S5. (a) The integration time for three integration events, with V_{DD} = 28 V. WL1 and WL2 are offset by 20 V and 40 V, respectively. As this is a two-bit system and the RS latch remains set after the second dose, the third pulse is again seen on WL2.

References

1. Smith, J. *et al.* Solution-Processed Small Molecule-Polymer Blend Organic Thin-Film Transistors with Hole Mobility Greater than 5 cm2/Vs. *Adv. Mater.* **24,** 2441–2446 (2012).

2. Anthony, J. E., Facchetti, A., Heeney, M., Marder, S. R. & Zhan, X. N-Type Organic Semiconductors in Organic Electronics. *Adv. Mater.* **22,** 3876–3892 (2010).

3. Mei, P. *et al.* Utilizing High Resolution and Reconfigurable Patterns in Combination with Inkjet Printing to Produce High Performance Circuits. *Appl. Phys. Lett.* **105,** 123301 (2014).
